# Deficiency of Epithelial PIEZO1 Alleviates Liver Steatosis Induced by High-Fat Diet in Mice

**DOI:** 10.7150/ijbs.102906

**Published:** 2025-01-01

**Authors:** Zhiyue Xu, Shu Xu, Xiaoming Liu, Lan Cheng, Xinghuang Liu, Xiaotian Xie, Dan Zhou, Dongke Wang, Jie Chen, Xiaoling Deng, Lei Zhang, Ruohang He, Ying Li, Mengmeng Cheng, Ling Yang, Xiaohua Hou, Tao Bai

**Affiliations:** 1 Division of Gastroenterology, Union Hospital, Tongji Medical College, Huazhong University of Science and Technology, Wuhan 430022, China; 2 Department of Radiology, Union Hospital, Tongji Medical College, Huazhong University of Science and Technology, Wuhan 430022, China.; 3 Department of Pathology, Union Hospital, Tongji Medical College, Huazhong University of Science and Technology, Wuhan 430022, China.

**Keywords:** PIEZO1, Gut-liver axis, Liver steatosis, FGF15/19, Retinol metabolism

## Abstract

PIEZO1 has been found to play a vital role in regulating intestinal epithelial cells (IEC) function and maintaining intestinal barrier in recent years. Therefore, IEC PIEZO1 might exert a significant impact on liver metabolism through the gut-liver axis, but there is no research on this topic currently. Classic high-fat diet (HFD) model and mice with IEC-specific deficiency of PIEZO1 (*Piezo1*^ΔIEC^) were used to explore the problem. IEC PIEZO1 deletion significantly alleviated liver steatosis, without change on glucose tolerance and energy expenditure. Fibroblast growth factor 15/19 (FGF15/19) was downregulated in IEC and portal vein of *Piezo1*^ΔIEC^ mice, which was associated with phenotypic change. After supplementary of exogenous FGF19, the effect of improving liver steatosis brought by PIEZO1 deletion was blocked. Notably, PIEZO1 depletion-induced FGF15 reduction was not dependent on classic bile acids (BAs) - farnesoid X receptor (FXR) pathway, but attributed to impaired retinol metabolism and lower content of retinoic acid (RA). Subsequently, addition of RA but not retinol benefited inducing FGF15 production in ileal organoid from *Piezo1*^ΔIEC^ mice. Altogether, IEC PIEZO1 represents a promising target for therapy of hepatic steatosis via the gut-liver axis.

## Introduction

Metabolic dysfunction-associated steatotic liver disease (MASLD), now was chosen to replace Non-alcoholic fatty liver disease (NAFLD), emphasize the impact of metabolic disorders^1^. The global prevalence of MASLD is as high as 30%, making it the most common chronic and progressive liver disease worldwide^2^. Heterotopic deposition of lipids can induce liver cell damage, leading to metabolic dysfunction-associated steatohepatitis (MASH), which would progress to cirrhosis, liver cancer, and end-stage liver disease over time^3^. Although some medications showed benefits for MASLD, there are currently no approved drugs for the treatment of MASLD at any disease stage^4^.

Substantial evidence in the past years supported a vital role of the gut-liver axis played in MASLD^5^. Impaired barrier function and disrupted bile acids (BAs) metabolism were two main intestinal factors causing liver injury and accelerating MASLD progress. The former worked through promoting the translocation of bacterial and harmful microbial metabolites such as ethanol and microbial related molecular patterns, while the latter by inducing abnormal intestinal farnesoid X receptor (FXR) signal and subsequent enterohepatic circulation of BAs^5^-^7^. Microbiota modulators such as probiotics, and BAs or derivates thus were tried to treat MASLD. However, results from large number of clinical trials revealed microbiota modulators can only work as an adjunct therapy^8^, while BAs or derivates showed multiple dose-dependent adverse effects including pruritis and other metabolic abnormality such as hyperlipidemia^9^. Intestinal epithelial cells (IEC) were the core of maintaining barrier function and BAs signaling transduction. Therefore, new targets in intestine especially IEC were needed to develop.

PIEZO1 is a non-selective cation channel, which has been found playing a noteworthy role in maintaining normal IEC physiological function^10^, ^11^. PIEZO1 maintains homeostatic epithelial cell number via mediating cell extrusion induced by overcrowding^12^. We previously also found PIEZO1 was closely related to intestinal epithelial permeability by affecting the expression of tight junction proteins and mucus secretion in IEC^10^, ^11^. Besides, recent studies reported PIEZO1 was involved in the metabolic homeostasis of certain tissues including adipocytes, pancreatic islets β and vascular endothelial cells. Targeted deletion of PIEZO1 in adipocytes and pancreatic islets β, can induce impaired insulin sensitivity and glucose tolerance, while PIEZO1 deficiency in vascular endothelial cells improved atherosclerosis^13^-^15^, which suggested the contributions of PIEZO1 to metabolic homeostasis might differ due to the tissue type where PIEZO1 in. On the whole, IEC PIEZO1 could candidate regulate liver metabolic homeostasis through the gut-liver axis.

Therefore, this study aimed to confirm the role of IEC PIEZO1 played in MASLD, and further explore potential mechanism based on the gut-liver axis.

## Methods

### Study design

Intestinal mucosa was gotten from human with or without MASLD to analyze whether IEC PIEZO1 was associated with MASLD. IEC-specific PIEZO1 deficiency mice were generated to determine the role of IEC PIEZO1 in MASLD development. Besides, energy intake and expenditure, lipid metabolism in IEC, intestinal barrier, microbiota and BAs were detected to explore the mechanism of IEC PIEZO1 affecting MASLD.

### Human study

Distal ileum mucosa samples were obtained from Union Hospital, Tongji medical college, Huazhong University of Science and Technology. Human related studies were approved by the Ethics Committee of Union Hospital (2020 [S092]) and all individuals signed informed consent before participating in the study. Each individual met the inclusion criteria: (1) fatty liver or non-fatty liver diagnosed by liver ultrasound; (2) no diseases including colorectal cancer, inflammatory bowel disease, acute or chronic viral hepatitis and alcoholic liver disease; (3) no pregnancy. Clinical characteristics of participants were summarized in Supplementary Table 1.

### Animals

Mice carrying flox alleles for PIEZO1 gene (abbreviated as *Piezo1*^fl/fl^) and Villin-Cre mice were constructed by Cyagen Biosciences (Suzhou, China), and used to generate mice with conditional knockout of PIEZO1 in the intestinal epithelium cells (abbreviated as *Piezo1*^ΔIEC^, Supplementary Fig. 1A-B). All mice were maintained in C57BL/6 genetic background. 6-8 weeks old, 22-25g male wild-type C57BL/6 mice were purchased from Vital River Laboratory Animal Technology Co., Ltd. (Beijing, China). All the mice were housed in a specific pathogen-free condition, maintained on a 12-h light/dark cycle, 22-26 degrees with free sterile food and water in the laboratory animal center of Huazhong University of Science and Technology, and acclimatized for at least one week before experiments. Animal experimental procedures were approved by the Institutional Animal Care and Use Committee of Huazhong University of Science and Technology (2023-4180).

The male mice were fed with a high-fat diet (HFD, 60 kcal% from fat, D12492, Research Diets, USA) or normal chow diet (NCD, 10 kcal% from fat; WQJX Bio-technology, China) for 12 weeks. For fibroblast growth factor 15/19 (FGF15/19) intervention, we used FGF19 because FGF15 is less stable. In general, FGF19 have the similar metabolic effects with FGF15 and have been utilized and FGF19 has been widely used in previous mouse studies^16^-^19^. Mice were fed with HFD for 8 weeks, followed by daily intraperitoneally injection with 50 μg/kg FGF19 (Abclonal, China) or 0.1% BSA during the rest 4 weeks of HFD feeding.

### Intraperitoneal glucose/insulin tolerance tests (IPGTT/IPITT)

Mice were fasted for 12 h prior to IPGTT and 6 h prior to IPITT. Mice were injected intraperitoneally with glucose solution (2 g/kg) or insulin (0.75 U/kg). Tail vein blood glucose concentrations were measured before (time 0) and at 15, 30, 60 and 120 min after glucose or insulin injection.

### Metabolic caging

Mice were individually housed in Comprehensive Lab Animal Monitoring System (Columbus Instruments, USA) for 48 hours before data collection. Food intake, volume of oxygen consumption (VO_2_) and carbon dioxide production (VCO_2_) were recorded. Respiratory exchange ratio (RER) was calculated by VCO_2_/VO_2_, heat production (kcal/kg/min) was calculated as (3.815 + 1.232 × RER) × VO_2_ and adjusted by lean mass.

### Liver MRI scan and body composition analysis

MRI examinations were performed on a 3.0 T MR imaging system (Philips Healthcare, Netherlands). Each mouse was fasted for 4 hours and then anaesthetized with an intraperitoneal injection of pentobarbital (1% w/v at 0.1ml/15g body weight) before imaging. The mice were placed in head-first prone position during the examination. To obtain experimental images of the liver, a coronal three-dimensional 6-echo mDixon-Quant gradient echo sequence was applied.

Transverse section was reconstructed by image post-processing workstation. The automatically generated proton density fat fraction (PDFF) images were used for measurement. Three regions of interest (ROIs) (area: 4 mm^2^) were drawn to calculate the mean PDFF value of the mouse liver. All measurements were executed independently by two radiologists (Liu Xiaoming, Cheng Lan), and the average value of the measurements was taken.

The body composition analyzer minispec LF50 (Bruker, German) was used to perform whole-body MRI scans on mice and measure fat mass and lean mass.

### Biochemical assays

TG, TC, ALT and AST levels in serum or liver were measured using biochemical kits (Jiancheng Bioengineering, China). FGF15 and CYP7A1 were measured by using ELISA kits (Cusabio & Sabbiotech, China).

### Histological analysis

Liver and intestine tissues were fixed with 4% paraformaldehyde, embedded with paraffin, and stained by hematoxylin and eosin (H&E). OCT-embedded frozen liver or intestine sections were stained by oil red O (ORO) according to standard procedures. Images were obtained by optical microscope (Olympus, Japan). Two pathologists (Chen Jie, Deng Xiaoling) conducted independent blind MASLD activity scoring and the average value of scores was taken. The MASLD activity score was defined as the unweighted sum of the scores for steatosis, lobular inflammation, and ballooning, which was reported by David E. Kleiner and others^20^. Detailly, steatosis, with scores of 0 (none), 1 (<33% hepatocytes affected), 2 (34%-66% hepatocytes affected), and 3 (>66% hepatocytes affected); lobular inflammation, with scores of 0 (no foci), 1 (<2 foci), 2 (2-4 foci), and 3 (>4 foci); and ballooning degeneration, with scores of 0 (none), 1 (few), and 2 (many).

### FITC-Dextran 4 kDa (FD4) permeability

FD4 (Sigma, USA) at a dosage of 0.75mg/g of body was given to mice by oral gavage after fasting for 3 h. Blood samples were obtained from orbital venous plexus via capillary tubes at 4 h after gavage. Serum samples were diluted 1/10 in saline, and fluorescent intensity was measured at 490 nm/520 nm.

### RNA-seq analysis

Total RNA was extracted from the ileal epithelium using TRIzol^®^ Reagent according the manufacturer's instructions (Invitrogen, USA) and genomic DNA was removed using DNase I (Takara, Japan). RNA-seq transcriptome libraries were prepared following TruSeqTM RNA sample preparation Kit from Illumina (San Diego, USA). Paired-end libraries were sequenced by Illumina NovaSeq6000 sequencing (150bp*2, BIOZERON Co., Ltd, China).

Raw paired end reads were trimmed and quality controlled by Trimmomatic. Then clean reads were separately aligned to reference genome with orientation mode using hisat2 software. The quality assessment of these data was taken by qualimap_v2.2.1. Use htseq to count each gene reads.

To identify DEGs (differential expression genes) between different samples, the expression level for each gene was calculated using the fragments per kilobase of exon per million mapped reads (FRKM) method. R statistical package edgeR was used for differential expression analysis. The DEGs between samples were selected using the following criteria: the logarithmic of fold change was greater than 2 and the false discovery rate (FDR) should be less than 0.05. To understand the functions of the differentially expressed genes, KEGG pathway analysis were carried out. DEGs were significantly enriched in pathways when their Bonferroni-corrected P-value was less than 0.05.

### 16S rRNA gene sequencing

Microbial sequencing was conducted as described previously^21^. Briefly, Microbial DNA of cecal contents was extracted, then V3-V4 regions were amplified and sent for 16S rRNA gene sequencing on an Illumina MiSeq 2×300 bp platform (Metabo-Profile, China). After quality control, high-quality sequences were clustered into operational taxonomic units (OTUs) at 97% sequence similarity and assigned to database including Greengene and Silva128 for taxonomy classification.

### Targeted metabolomics of bile acids (BAs)

BAs in cecal contents were measured by using an ultra-performance liquid chromatography coupled to tandem mass spectrometry (UPLC-MS) system (Metabo-Profile, China). More details were provided in the Supplementary Material.

### Measurement of retinol and all-trans retinoic acid (atRA)

Retinol and atRA were detected an ultra-performance liquid chromatography coupled to tandem mass spectrometry (UPLC-MS) system (Metabo-Profile, China). More details were provided in the Supplementary Material.

### IEC isolation

Ileum was longitudinally opened, rinsed with cold PBS, diced into 2-3mm long pieces and incubated in 8 mM EDTA on ice for 60 minutes. The pieces were then transferred to centrifuge tube containing PBS and shaken violently to make epithelial cells fall off. Then the IEC was obtained from PBS containing large number of epithelial cells after centrifugation.

### Organoid culture and treatment

Crypts isolation and organoid culture were conducted as described previously^22^. Briefly, cleaned small intestine sections was incubated in 2.5 mM EDTA at 4°C for 40 min, and then shaken vigorously in cold PBS containing 0.1% BSA. After passing through 70μm cell strainers, crypt fractions were isolated via centrifugation at 290g for 5 minutes. 500 crypts per well were seeded in 24-well plates followed by suspending with 1:1 IntestiCult™ Organoid Growth Medium (Stemcell Technologies, Canada) and Matrigel (Corning, USA). Extra medium was added after the domes solidified. For intervention of retinol metabolic pathway, medium containing 1µM retinol or 0.1µM atRA (Sigma, USA) was added to the wells for 72h.

### Real-time quantitative polymerase chain reaction (RT-qPCR)

Total RNA was isolated with RNA-easy^TM^ Isolation Reagent (Vazyme, China). The concentration of total RNA was equilibrated after the concentration and purity were determined by Nanodrop 2000 Spectrophotometer (ThermoScientific, USA). HiScript III All-in-one RT SuperMix Perfect for qPCR (Vazyme, China) was used to make RNA reverse transcribed to complementary DNA (cDNA). Mixture of cDNA, SYBR Green (ThermoScientific, USA) and Primers (Supplementary Material 2) was allowed to react and fluorescence signal was detected by LightCycler480 (Roche Diagnostics, Switzerland) to determine gene expression changes. *Gapdh/GAPDH* was used as an internal control.

### Western blot

Proteins were extracted with RIPA Lysis Buffer (Beyotime, China) containing 1% PMSF (Beyotime, China). Protein concentrations were measured by bicinchoninic acid (BCA) assay kit (Boster, China). Denatured protein samples with same protein amount were separated by SDS-PAGE (Epizyme, China) and then transferred onto PVDF membranes (Millipore, USA). Membranes were blocked with NcmBlot Blocking Buffer (NCM Biotech, China) for 15 minutes, and the incubated with antibody against β-ACTIN (1:10000, Proteintech, China), PIEZO1 (1:1000, 15939-1-AP, Proteintech, China), ZO1 (1:1000, 40-2200, Invitrogen, USA), OCCLUDIN (1:1000, 710192, Invitrogen, USA), FXR (1:1000, 25055-1-AP, Proteintech, China), SHP (1:1000, YN0999, Immunoway, USA) at 4℃ overnight. After the membranes were washed with TBST thrice for 10 min each time, they were incubated with HRP-conjugated goat anti-rabbit or anti-rabbit secondary antibodies (1:5000, SA00001-1/SA00001-2, Proteintech, China). Protein blots were visualized by enhanced ECL kit (NCM Biotech, China) and subjected to LAS-4000 imaging system (Fujiflim, Japan).

### Statistical analysis

Data were reported as mean ​± ​standard error (SEM). Differences between groups was determined by Student t test (two groups), one-way ANOVA with Bonferroni post hoc test. Two-sided tests were applied in all statistical tests, and a P value less than 0.05 was considered statistically significant. All statistical tests were performed by the GraphPad Prism version 9.4 software (La Jolla, USA).

## Results

### PIEZO1 was downregulated in intestine epithelium of humans and mice with fatty liver

**Figure 1:**
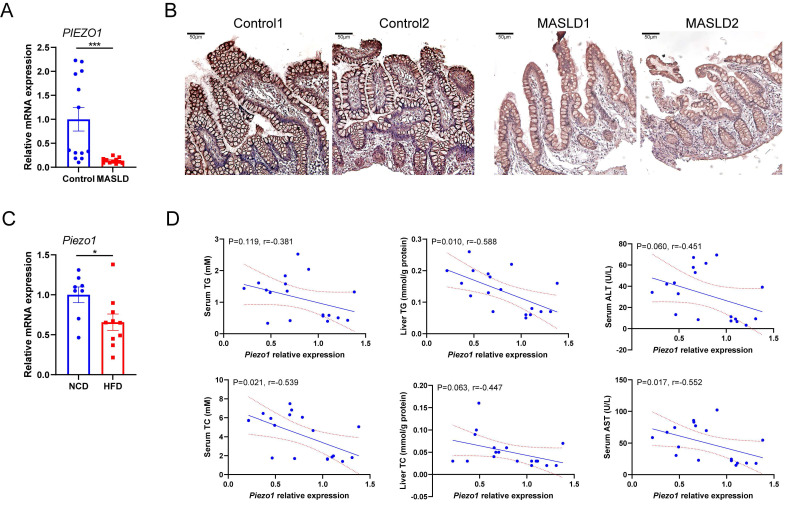
** PIEZO1 was downregulated in intestine epithelium of humans and mice with fatty liver.** (A) mRNA levels of *PIEZO1* in ileum biopsies from individuals with MASLD (n=11) or without MASLD (Control, n=13). (B) Representative immunohistochemical staining of PIEZO1 in human ileum biopsies, scale bar: 50µm. (C) mRNA levels of *Piezo1* in IEC from mice fed with HFD (n=10) or NCD (n=8) for 12weeks. (D) Correlation of IEC *Piezo1* relative mRNA expression with serum TG/TC, liver TG/TC, and serum ALT/AST levels in mice (n=18). Data are presented as mean ± sem. ^*^P < 0.05, **P < 0.01, ***P < 0.001.

Association between intestinal PIEZO1 and liver steatosis was evaluated by detecting PIEZO1 expression in distal ileum mucosa from individuals with or without fatty liver. Compared to control persons, lower PIEZO1 expression was observed in humans with fatty liver (Fig. 1A, B). Consistent with the human data, *Piezo1* mRNA expression in IEC were significantly decreased after a 12-week HFD in wild C57BL/6 mice (Fig. 1C), presented a negative correlation with serum TC, liver TG, serum AST and a negative correlation trend with serum TG, liver TC, serum ALT (Fig. 1D).

### PIEZO1 deficiency in IEC alleviated HFD-induced liver steatosis of mice

To investigate the role of IEC PIEZO1 played in the development of MASLD, *Piezo1*^fl/fl^ mice and IEC-specific *Piezo1*-knockout (*Piezo1*^ΔIEC^) mice were used. mRNA and protein expression levels were significantly decreased in IEC, with no changes in liver of *Piezo1*^ΔIEC^ mice (Supplementary Fig. 1C, D).

**Figure 2:**
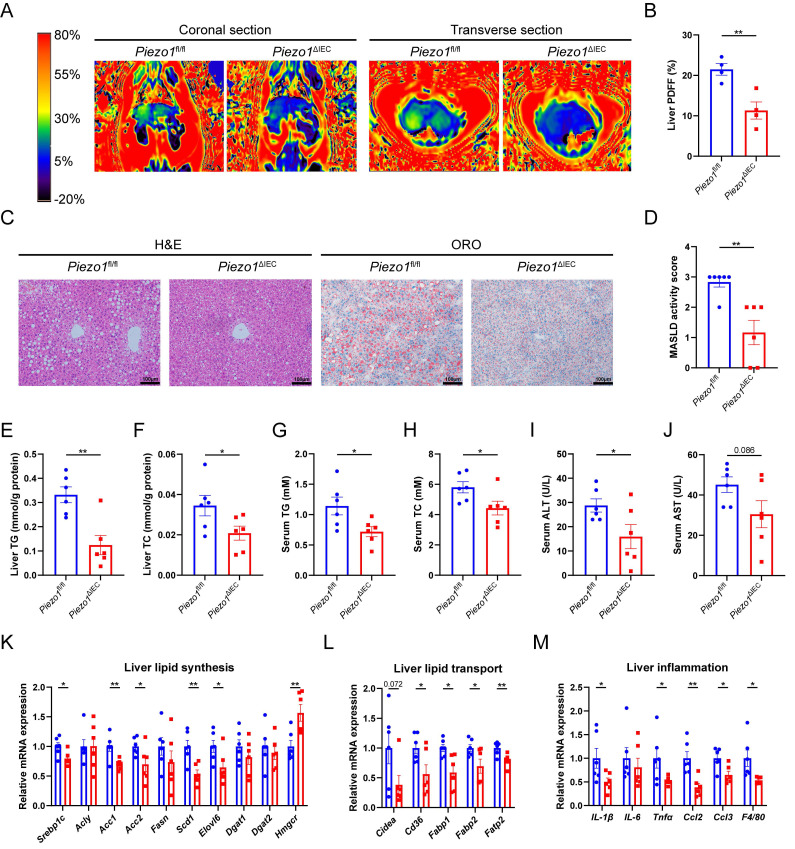
** PIEZO1 deficiency in IEC alleviated HFD-induced liver steatosis of mice.** (A) Representative coronal and transverse MRI images, (B) Proton density fat fraction (PDFF) in MRI (n=4/group), (C) Representative liver H&E and ORO staining images, scale bar: 100µm, (D) MASLD activity score, (E-J) Liver TG/TC, serum TG/ TC and serum ALT/AST level, (K-M) mRNA levels of genes associated with lipid synthesis, lipid transport and inflammation in liver, of *Piezo1*^fl/fl^ and *Piezo1*^ΔIEC^ mice after feeding with HFD for 12 weeks, n=6.group. Data are presented as mean ± sem. ^*^P < 0.05, **P < 0.01, ***P < 0.001.

*Piezo1*^fl/fl^ and *Piezo1*^ΔIEC^ mice were fed with a HFD for 12 weeks. Compared to *Piezo1*^fl/fl^ mice, *Piezo1*^ΔIEC^ mice showed a significantly weakened intrahepatic fat signal in MRI (Fig. 2A, B), less and smaller lipid droplets in H&E and ORO staining (Fig. 2C, D). Furthermore, *Piezo1*^ΔIEC^ mice displayed significantly lower TG and TC levels of liver and serum, lower serum ALT and a decreasing trend of AST levels reflecting hepatic lipotoxicity (Fig. 2E-J).

The mRNA expression of genes associated with lipid synthesis and transport in liver were reduced in *Piezo1*^ΔIEC^ mice as compared to *Piezo1*^fl/fl^ mice (Fig. 2K, L). After 12-week HFD intake, there was no obvious inflammatory cell infiltration among all mice, but mRNA expression levels of some inflammatory cytokines and chemokines, including *IL-1β*, *Tnfα*, *Ccl2*, *Ccl3* and *F4/80* were markedly decreased in livers of *Piezo1*^ΔIEC^ mice (Fig. 2M).

However, intestinal PIEZO1 deficiency did not affect hepatic steatosis under a normal chow diet (Supplementary Fig. 2). In addition, *Piezo1*^ΔIEC^ mice did not exhibited significant improvements in body weight, fat mass, blood glucose, insulin sensitivity and the whole-body energy metabolism (Supplementary Fig. 3).

### PIEZO1 ablation downregulated FXR-FGF15/19 signaling pathway in IEC and reduced FGF15 secretion

Intake, absorption, synthesis and output of dietary fat in intestine were firstly examined and similar between two genotypes. Besides, intestinal villus morphology and barrier function was next assessed for its close association with MASLD, in which *Piezo1*^ΔIEC^ mice did not differ from *Piezo1*^fl/fl^ mice (Supplementary Fig. 4).

Past studies confirmed that FXR-FGF15/19 signaling pathway played important roles in the gut-liver crosstalk associated with MASLD^23^. Therefore, we analyzed whether FXR-FGF15/19 signaling pathways changed in the intestine and liver in *Piezo1*^ΔIEC^ mice.

**Figure 3:**
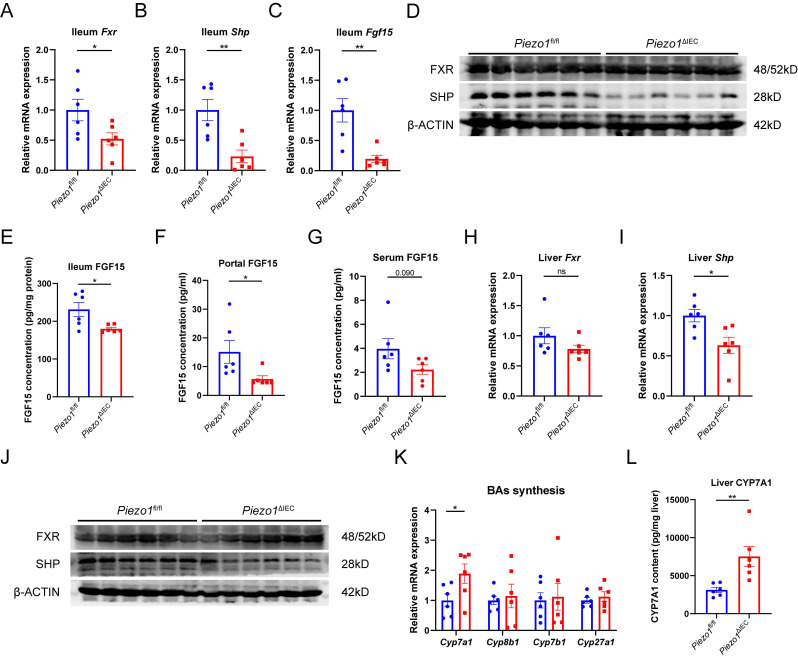
** PIEZO1 ablation downregulated FXR-FGF15/19 signaling pathway in IEC and reduced FGF15 secretion.** mRNA levels of (A) *Fxr*, (B) *Shp* and (C) *Fgf15* in ileum epithelium, n=6/group. (D) Western blot of FXR and SHP in ileum epithelium, n=6/group. (E) FGF15 concentration in ileum epithelium, n=6/group. (F) FGF15 concentration in portal vein, n=6/group. (G) FGF15 concentration in serum, n=6/group. mRNA levels of (H) *Fxr*, (I) *Shp* in liver, n=6/group. (J) Western blot of FXR and SHP in liver, n=6/group. (K) mRNA levels of genes associated with BAs synthesis in liver, n=6/group. (L) CYP7A1 protein level in liver by ELISA, n=6/group. Data are presented as mean ± sem. ^*^P < 0.05, **P < 0.01, ***P < 0.001. ns, not significant.

In ileum epithelium, mRNA expression levels of *Fxr* and its targeted gene (*Shp* and *Fgf15*) were all substantially decreased in *Piezo1*^ΔIEC^ mice compared to *Piezo1*^fl/fl^ mice (Fig. 3A-C). Differently, SHP and FGF15 of ileum epithelium, and portal FGF15 were coherently reduced in protein level but FXR did not (Fig. 3D-G), suggesting that change of FGF15 could possibly be dependent on FXR activity. Meanwhile, along with reduction of FGF15, SHP in liver was downregulated while CYP7A1 was upregulated (Fig. 3H-L).

### Exogenous FGF19 exacerbated liver steatosis in HFD-fed *Piezo1*^ΔIEC^ mice

**Figure 4:**
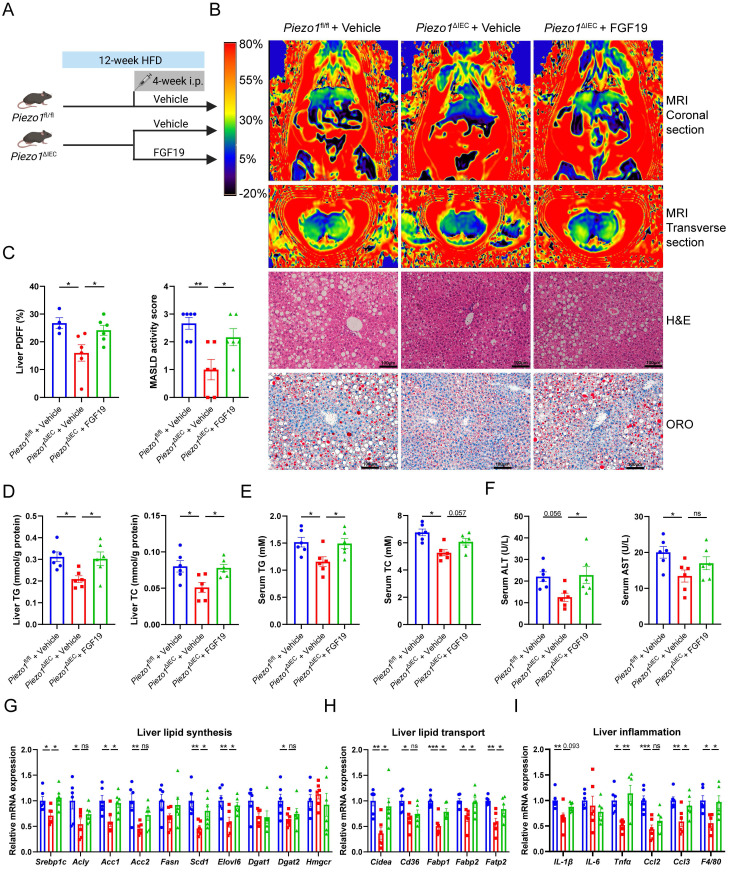
** Exogenous FGF19 exacerbated liver steatosis in *Piezo1*^ΔIEC^ mice.** (A) Schematic of the treatment protocol for mouse treatment with vehicle or FGF19 for 4 weeks. (B) Representative liver coronal and transverse MRI, H&E and ORO staining images, (C) Proton density fat fraction (PDFF) in MRI (left, n=4-6/group), and MASLD activity score (right, n=6/group), (D-F) Liver TG/TC, serum TG/ TC and serum ALT/AST level, (G-I) mRNA levels of genes associated with lipid synthesis, lipid transport and inflammation in liver, of 3 group mice after feeding with HFD for 12 weeks, n=6/group. Data are presented as mean ± sem. ^*^P < 0.05, **P < 0.01, ***P < 0.001. ns, not significant.

To verify whether downregulation of FGF15 could explain why PIEZO1 knockout in IEC alleviated HFD-induced liver steatosis, exogenous recombinant human FGF19 protein was supplemented to HFD-fed *Piezo1*^ΔIEC^ mice for 4 weeks (Fig. 4A).

Supplementary of FGF19 reversed the intrahepatic fat signal of MRI, caused more and larger lipid droplets in liver of *Piezo1*^ΔIEC^ mice (Fig. 4B, C), but without change in weight of body or adipose tissues (Supplementary Fig. 5). Compared with that of vehicle-treated *Piezo1*^ΔIEC^ mice, hepatic TG and TC levels and serum TG levels were increased in FGF19-treated *Piezo1*^ΔIEC^ mice (Fig. 4D-F).

Similar to the aggravation of liver steatosis, mRNA expression levels of genes associated with lipid synthesis, lipid transport and inflammation were increased in the liver of FGF19-treated *Piezo1*^ΔIEC^ mice (Fig. 4G-I).

### Downregulation of FXR-FGF15/19 in *Piezo1*^ΔIEC^ mice was independent from microbiota-BAs metabolism

**Figure 5:**
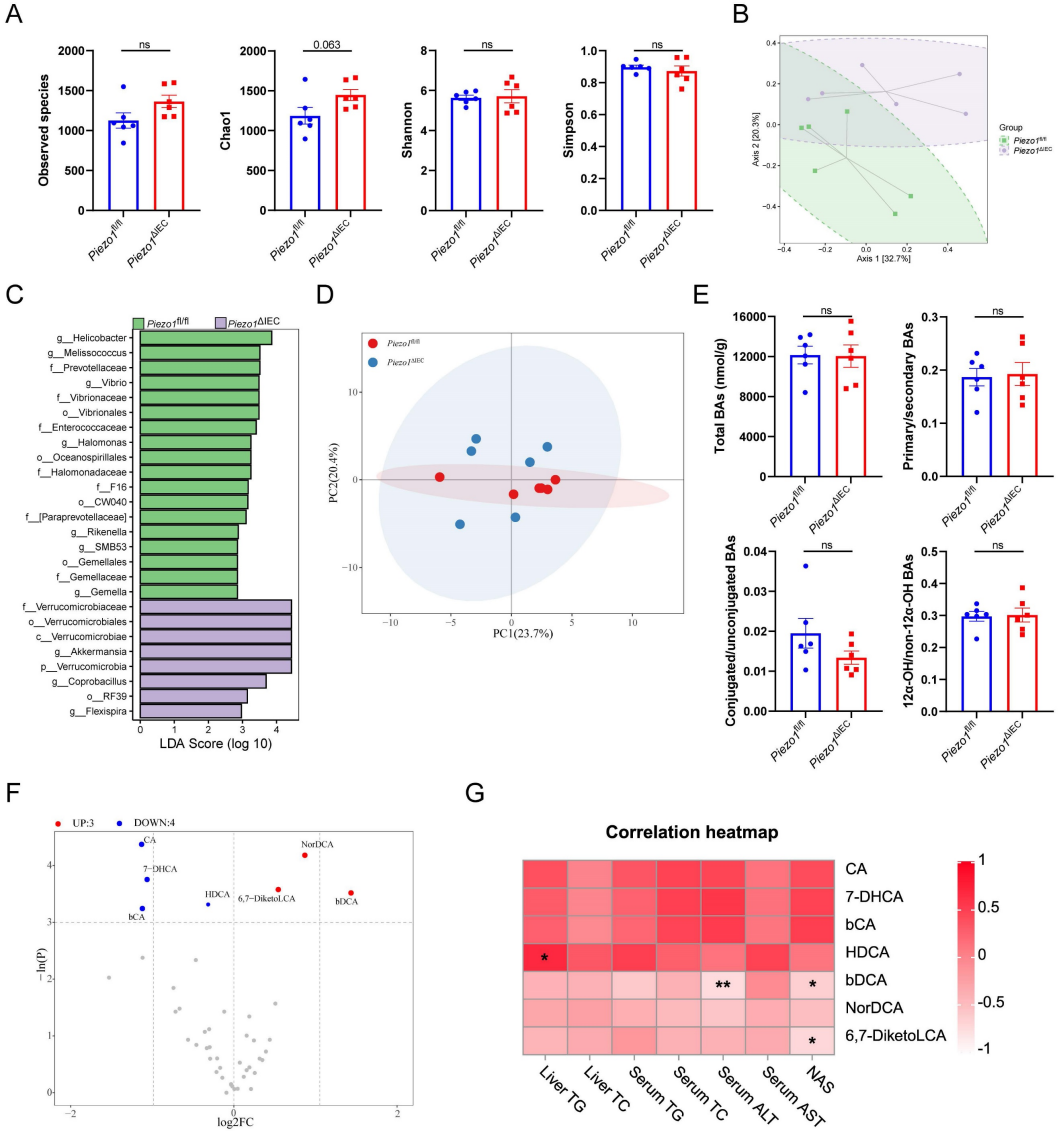
** Downregulation of FXR-FGF15/19 in *Piezo1*^ΔIEC^ mice was independent from microbiota-BAs metabolism.** (A) 4 alpha diversity indexes, (B) PCoA plot based on Bray-Curtis distance, and (C) Enriched bacteria by LEfSe of fecal microbiota in *Piezo1*^fl/fl^ and *Piezo1*^ΔIEC^ mice after feeding with HFD for 12 weeks, n=6/group. (D) PCA of total BAs composition. (E) Total BAs contents, proportion of primary/secondary BAs, proportion of conjugated/unconjugated BAs and 12α-OH/non-12α-OH BAs, and (F) 7 kinds of BAs significantly changed, in cecal contents of *Piezo1*^fl/fl^ and *Piezo1*^ΔIEC^ mice after feeding with HFD for 12 weeks, n=6/group. (G) Correlation of 7 kinds of BAs with index about lipid level or liver damage, n=12. Data are presented as mean ± sem. ^*^P < 0.05, **P < 0.01, ***P < 0.001. ns, not significant.

Given the important regulatory role of microbiota-BAs metabolism played in FXR-FGF15/19 signaling pathway^24^, microbial sequencing and BAs targeted metabolomics were perform on cecal contents. The 16S rRNA gene sequencing showed that there was no change in alpha diversity of intestinal microbiota between *Piezo1*^ΔIEC^ mice and *Piezo1*^fl/fl^ mice (Fig. 5A). However, the beta diversity refers to microbiota composition of *Piezo1*^ΔIEC^ mice obviously differed from that of *Piezo1*^fl/fl^ mice by principal coordinate analysis (Fig. 5B, PermANOVA test P=0.048). Besides, LEfSe analysis was employed to identify differential bacteria. Some abundance-upregulated bacteria such as *Akkermansia* and abundance-downregulated bacteria such as *Rikenella* were discovered in *Piezo1*^ΔIEC^ mice (Fig. 5C). However, there was no evidence to suggest these bacteria are involved in intestinal BAs metabolism^25^.

Overall, the BAs profiles of cecal contents between *Piezo1*^ΔIEC^ mice and *Piezo1*^fl/fl^ mice were similar (Fig. 5D). No matter in total BAs level, proportion of primary/secondary BAs, proportion of conjugated/unconjugated BAs nor proportion of 12α-OH/non-12α-OH BAs, BAs composition of HFD-fed *Piezo1*^ΔIEC^ mice did not differ from that of HFD-fed *Piezo1*^fl/fl^ mice (Fig. 5E). Both upregulated bile acids (NorDCA, βDCA, and 6,7-DiketoLCA) and downregulated bile acids (CA, βCA, 7-DHCA and HDCA), showed limited correlation with lipid levels (Fig. 5F, G). HDCA was confirmed that it could alleviate liver steatosis which benefited liver^26^, and CA has a very weak agonist effect on FXR^27^, both of which were not able to effectively explain the phenotype in this study.

### PIEZO1-depleted IEC exhibited weakened retinol metabolism, impairing FGF15 production

**Figure 6:**
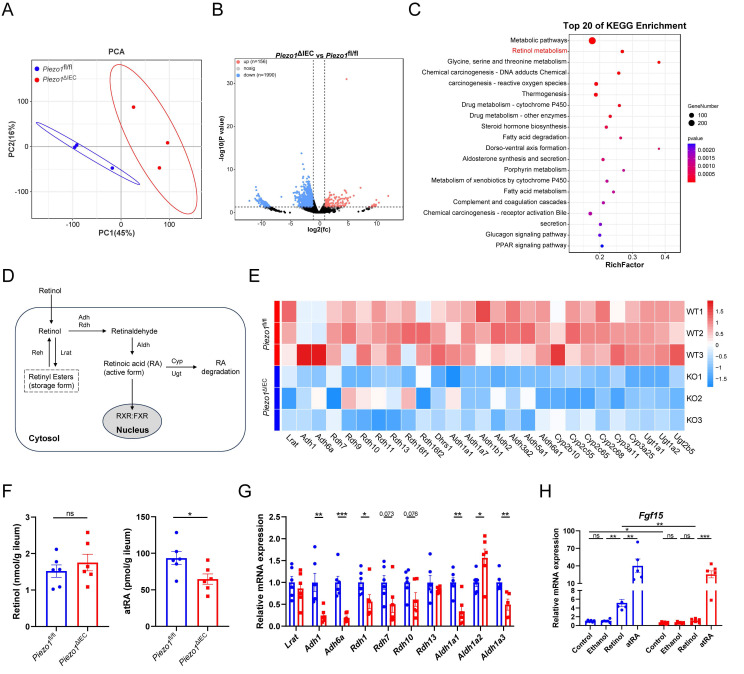
** PIEZO1-depleted IEC exhibited weakened retinol metabolism, impairing FGF15 production.** (A) PCA of gene expression, (B) Volcano plot depicting the differentially expressed genes, and (C) The top 20 most enriched KEGG pathways of differentially expressed genes, in ileum epithelium from *Piezo1*^fl/fl^ and *Piezo1*^ΔIEC^ mice fed with HFD for 12 weeks, n=3/group. (D) Schematic diagram of retinol metabolism focuses on RA synthesis from retinol. (E) The heat map of differentially expressed genes associated with retinol metabolism based on RNA-seq data of ileum epithelium from *Piezo1*^fl/fl^ and *Piezo1*^ΔIEC^ mice fed with HFD for 12 weeks, n=3/group. (F) Retinol and atRA content in ileum epithelium, n=6/group. (G) mRNA levels of partial genes associated with retinol metabolism in ileum organoids separated from *Piezo1*^fl/fl^ and *Piezo1*^ΔIEC^ mice fed with HFD for 12 weeks and cultured for 7 days, n=5-6/group. (H) mRNA levels of *Fgf15* in ileum organoids from *Piezo1*^fl/fl^ and *Piezo1*^ΔIEC^ mice fed with HFD for 12 weeks and treated with ethanol, retinol, or atRA for 3 days, n=5-6/group. Data are presented as mean ± sem. ^*^P < 0.05, **P < 0.01, ***P < 0.001. ns, not significant.

To further explore if there were other factors resulting in a decrease in FGF15 production, RNA-sequency of IEC was employed. The results of PCA showed that genes expression profile of IEC in the *Piezo1*^ΔIEC^ mice was significantly different from that in *Piezo1*^fl/fl^ mice, with a total of 156 genes upregulated and 1990 genes downregulated (Fig. 6A, B). Notably, the KEGG pathway enrichment analysis revealed that differentially expressed genes between ileum IEC of *Piezo1*^ΔIEC^ mice and *Piezo1*^fl/fl^ mice were significantly enriched in retinol metabolism (Fig. 6C).

Retinoic acid (RA), a main metabolite of retinol, can also effectively regulate FGF15 expression through retinoid X receptor (RXR), which can form a heterodimer with FXR^24^. Moreover, genes involved in the biosynthesis of RA from retinol, such as *Adh1*, *Adh6a*, *Aldh1a1*, and *Aldh1a3*, were downregulated in the IEC of *Piezo1*^ΔIEC^ mice compared with *Piezo1*^fl/fl^ mice (Fig. 6D, E).

The amount of retinol and RA were then quantified. Lower content of atRA, but not retinol, was founded in the IEC of *Piezo1*^ΔIEC^ mice (Fig. 6F). Besides, small intestinal organoids were used to further exam the role of retinol metabolism in PIEZO1-regulated FGF15 expression. Organoids of *Piezo1*^ΔIEC^ mice showed wide downregulation in genes related to transition from retinol to RA and lower *Fgf15* mRNA level (Fig. 6G, H). Both retinol and atRA activated *Fgf15* expression in organoids of *Piezo1*^fl/fl^ mice, while only atRA elevated *Fgf15* expression in *Piezo1*^ΔIEC^ mice (Fig. 6H).

## Discussion

In the present study, IEC-specific deficiency of PIEZO1 alleviated HFD-induced liver steatosis, without change on glucose and energy metabolism, providing us with a new target for MASLD treatment. We next confirmed that reduced synthesis of FGF15 rather than improved intestinal barrier function mediated the phenotype change of *Piezo1*^ΔIEC^ mice, which suggested specific function of IEC played a prominent role in liver lipid metabolism. Notably, PIEZO1 depletion-induced FGF15 reduction was not dependent on classic BAs-FXR pathway, but attributed to impaired retinol metabolism and lower content of RA.

The effect of IEC PIEZO1 deficiency on liver steatosis was liver-targeted to a great extent, because there was no significant change in whole-body metabolism and fat distribution. Usage of IEC-specific PIEZO1 knockout mice and application of both stereoscopic imaging of liver fat based on MRI and traditional staining of lipid droplet, made the results to be solid. In fact, little is known about the relationship between PIEZO1 and lipid metabolism up to now. Limited studies reported PIEZO1 in other tissues were involved in metabolic homeostasis but not focused on liver lipid metabolism^13^, ^14^, ^28^. Adipocytes-specific deletion of PIEZO1 in mice resulted larger adipocytes and impaired insulin sensitivity by inhibiting differentiation of preadipocyte into mature adipocytes^13^. Impaired glucose tolerance and reduced glucose-induced insulin secretion were appeared in β-cell-specific PIEZO1 knockout mice because of decreased β-cell electrical activity and Ca^2+^ elevation^15^. By contrast, specific disruption of PIEZO1 in the vascular endothelium prevents progression of atherosclerosis^28^. On the whole, the contributions of PIEZO1 to metabolic homeostasis especially lipid or glucose metabolism might differ due to the tissue type where PIEZO1 in, and we focused IEC PIEZO1 here.

Among the mechanisms affecting liver lipid metabolism associated with gut-liver axis, we thought FGF15 reduction was the core mechanism of MASLD improvement, because energy intake and expenditure, lipid metabolism in IEC and intestinal barrier were detected and excluded. Notably, except for ghrelin^29^, gastrin^30^ and 5-HT^31^, we firstly pointed out PIEZO1 can also regulate another gastrointestinal-derived hormone FGF15. Although the role and mechanism of FGF15/19 played in liver steatosis was still ambiguous^32^-^35^, we confirmed that FGF15 reduction mediated the amelioration of liver steatosis caused by PIEZO1 deficiency by applying FGF19 supplementary experiment in HFD-fed *Piezo1*^ΔIEC^ mice.

FGF15/19 signaling exerted biological effects in the liver mainly by targeted regulating CYP7A1^24^, ^36^. In line with previous studies, we noted that liver SHP was downregulated and CYP7A1 was upregulated, which were consistent with the decrease in intestinal and portal FGF15. CYP7A1 deficiency in human leaded to a hyperlipidemic phenotype^37^, and upregulation of CYP7A1 can greatly decrease blood lipids levels^38^. Similarly, overexpression of CYP7A1 in a mice MASLD model effectively alleviated hepatic steatosis via activation of the hepatic BAs signaling pathway, inhibition of lipid synthesis, and decreased fatty acid uptake^39^-^41^, which was consistent with the downregulation of genes related to fatty acid transport and synthesis in *Piezo1*^ΔIEC^ mice liver in this study.

Impaired retinol metabolism and reduction of RA was the way of PIEZO1 deficiency downregulating FGF15. Considering that FXR often formed heterodimer with RXR^42^, ^43^, no change in FXR protein expression and slight change in BAs firstly reminded us that PIEZO1 deletion-induced reduction of FGF15 was possibly attributed to blocked RXR signal transduction. We next applied retinol and RA treatment on ileal organoid without PIEZO1, and confirmed that impaired retinol metabolism and reduction of RA mediated downregulation of FGF15, which was also reported in other studies^44^, ^45^. Totally, this study is the first to examine the regulatory role of PIEZO1 in retinol metabolism and FGF15 synthesis.

It is interesting that glucose and energy metabolism were not changed in *Piezo1*^ΔIEC^ mice. Genetic overexpression or pharmacologic administration of FGF15/19 increased energy expenditure and improved glucose tolerance^46^-^48^, suggesting that the regulation of lipid metabolism and glucose metabolism by FGF15/19 may be opposite. However, lateral ventricle injections of FGF15/19 exhibited an equivalent increase in the metabolic rate comparable to systemic administration^49^. Besides, KLB deficiency in the central nervous system but not knockout of FGFR4 or KLB in liver, blocked energy expenditure and glucose tolerance change brought by FGF15/19^48^, ^50^. These studies indicated that, like numerous gut-derived hormones^47^, ^51^, FGF15/19 affect energy expenditure and glucose tolerance by acting on the central nervous system rather liver or other peripheral organs. It was reported that influx of FGF15/19 into mouse brain was nonlinear and affected by blood concentration^52^. Meanwhile, hypothalamus can produce FGF15/19 to regulate hypothalamic FGF15/19 signaling to some extent^53^. Therefore, reduction of intestine and portal vein FGF15 in *Piezo1*^ΔIEC^ mice may not alter overall energy metabolism and glucose metabolism.

In conclusion, this study showed IEC PIEZO1 was downregulated in human and mice with MASLD, confirmed that genetic reduction of IEC PIEZO1 ameliorated liver steatosis via blocking retinol metabolism and FGF15 production, and highlighted IEC PIEZO1 as a promising target for therapy of hepatic steatosis via the gut-liver axis.

## Supplementary Material

Supplementary materials and methods, figures and table.
