# Supplementary material for: Deficiency of Epithelial PIEZO1 Alleviates Liver Steatosis Induced by High-Fat Diet in Mice
Source: Int J Biol Sci. 2025 Jan 1;21(2):745–57. doi: 10.7150/ijbs.102906 (PMC11705646; doi:10.7150/ijbs.102906)
Supplement: Supplementary file 1 — Supplementary materials and methods, figures and table. [file ijbsv21p0745s1.pdf]

Supplementary Material 1

Supplementary Table 1. Demographic characteristics of the subjects

Characteristics	Control	MASLD	P value
Gender, n			
Male	7	9	0.211[a]
Female	6	2	
Age, years			
Mean (sd)	59.92 (6.60)	54.27 (7.90	0.069[b]
Body weight, kg			
Mean (sd)	62.88 (11.07)	78.77 (12.43)	0.003[b]
Body mass index, kg/m^2			
Mean (sd)	23.17 (2.41)	26.70 (3.18)	0.005[b]

a: Fisher's precision probability test

b: Student’s t test

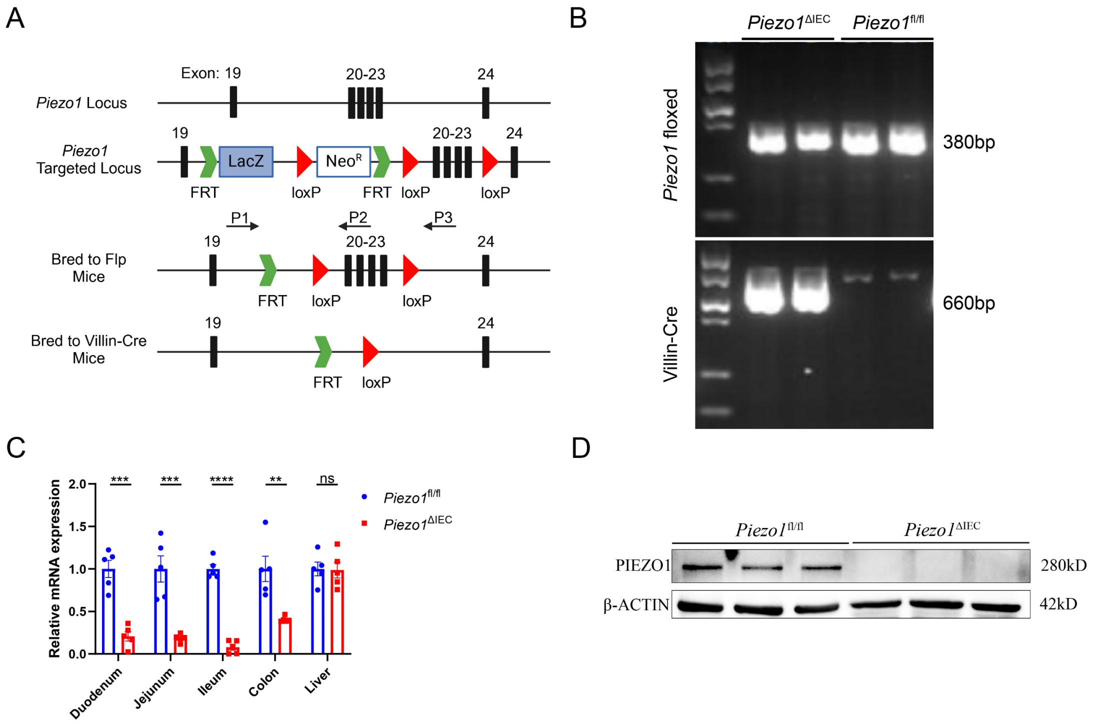

Supplementary Figure 1. Construction and validation of *Piezo1*$^{\Delta IEC}$ mice

(A) Schematic diagram of construction. (B) DNA identification. (C) mRNA level of *Piezo1* in duodenum, jejunum, ileum, colon and liver, n=5/group. (D) Western blot of PIEZO1 in ileum epithelium, n=3/group.

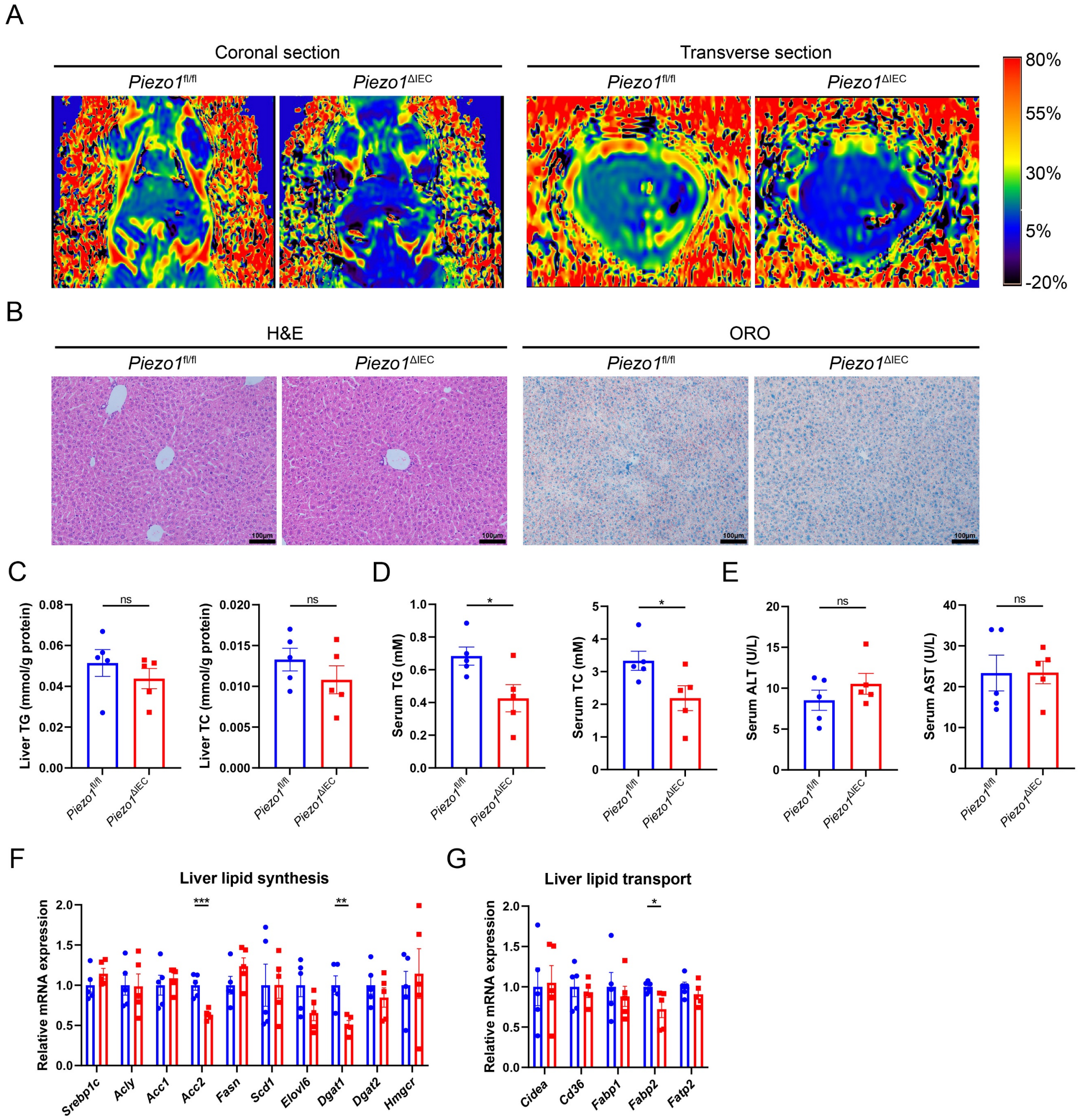

Supplementary Figure 2. PIEZO1 deficiency in IEC did not affect liver phenotype on an NCD

(A) Representative MRI images at coronal and transverse sections, (B) Representative liver H&E and ORO staining images, (C-E) Liver TG/TC, serum TG/ TC and serum ALT/AST level, (F-G) mRNA levels of genes associated with lipid synthesis, lipid transport and inflammation in liver, of *Piezo1*$^{fl/fl}$ and *Piezo1*$^{\Delta IEC}$ mice after feeding with HFD for 12 weeks, n=5/group. Data are presented as mean ± sem. *P < 0.05, **P < 0.01, ***P < 0.001. ns, not significant.

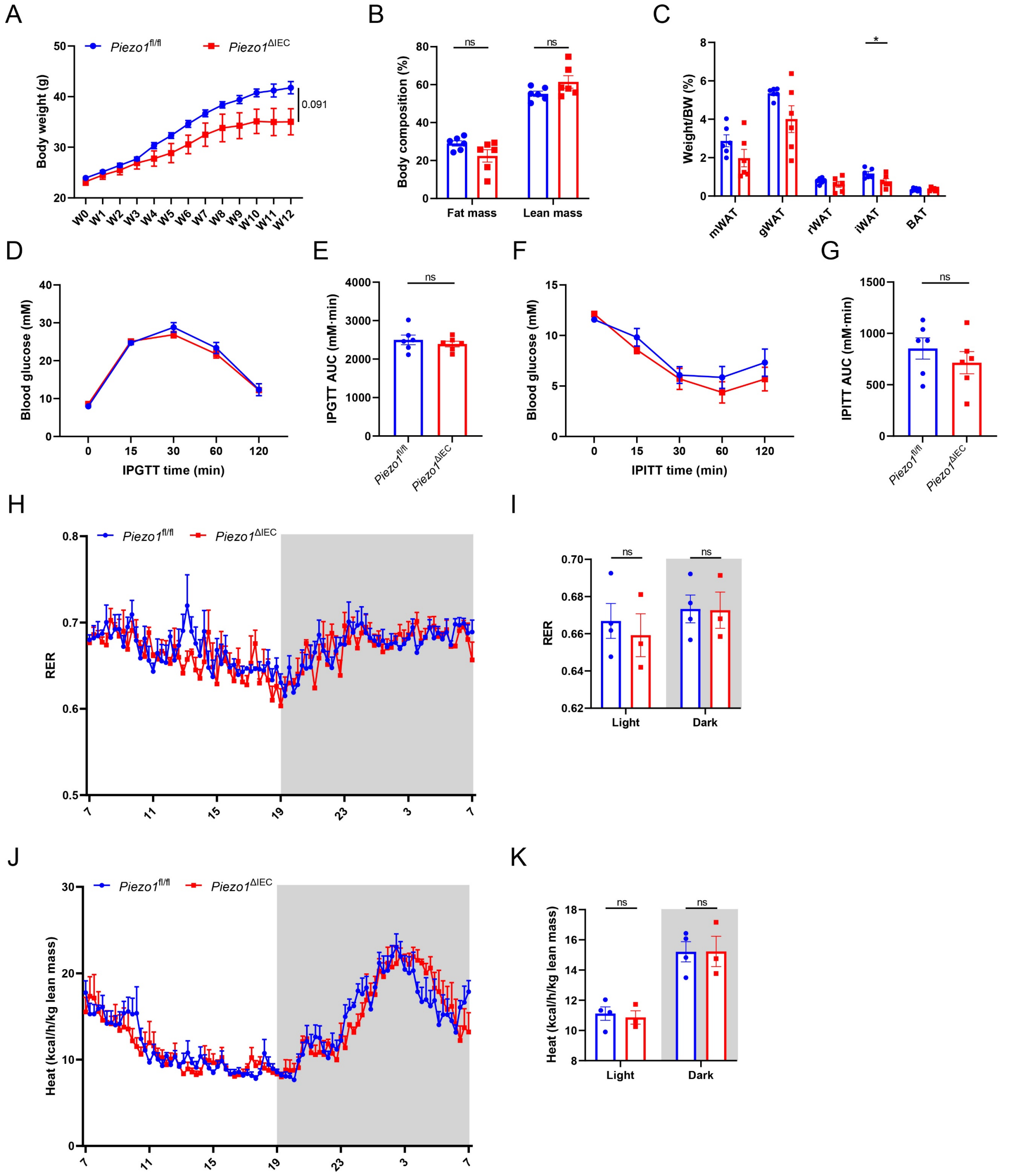

Supplementary Figure 3. PIEZO1 deficiency did not affect glucose and energy metabolism

(A) Body weight during 12-week HFD. (B) Body composition by MRI, (C) Relative weight of white adipose tissue (mesentery, gonad, renal and inguen) and brown adipose tissue, (D) IPGTT, (E) IPGTT AUC, (F) IPITT, (G) IPITT AUC of *Piezo1*$^{fl/fl}$ and *Piezo1*$^{\Delta IEC}$ mice after feeding with 12-week HFD, n=6/group. (H-I) Respiratory exchange ratio (RER), (J-K) Heat production of *Piezo1*$^{fl/fl}$ and *Piezo1*$^{\Delta IEC}$ mice fed with HFD for 11 weeks, n=3-4/group. Data are presented as mean ± sem. *$P < 0.05$, **$P < 0.01$, ***$P < 0.001$. ns, not significant.

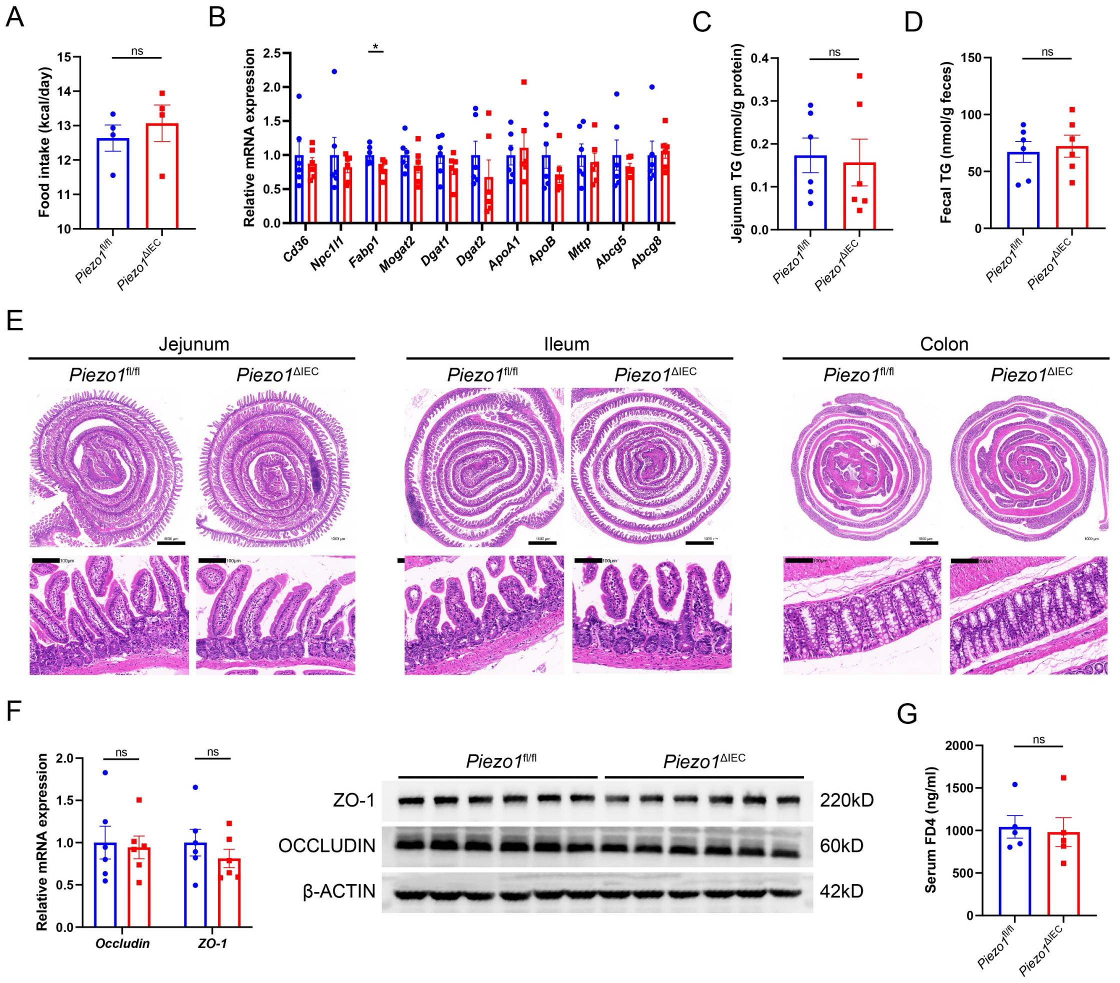

Supplementary Figure 4. *Piezo1*$^{\Delta IEC}$ mice did not alter intestinal lipid absorption, synthesis and barrier function

(A) Food intake (n=4/group). (B) mRNA levels of genes associated with lipid absorption, synthesis and output in jejunum, (C) Jejunum TG content, (D) Fecal TG content, n=6/group. (E) Representative jejunum, ileum and colon H&E staining images of *Piezo1*$^{fl/fl}$ and *Piezo1*$^{\Delta IEC}$ mice after feeding with 12-week HFD, scale bar: 1000μm, 100μm. (F) mRNA levels (left, n=6/group) and western blot image (right, n=3/group) of Occludin and ZO-1. (G) Serum FD4 concentration at 4 h after FD4 gavage in 0.75mg/g of body, n=5/group. Data are presented as mean ± sem. *P < 0.05, **P < 0.01, ***P < 0.001. ns, not significant.

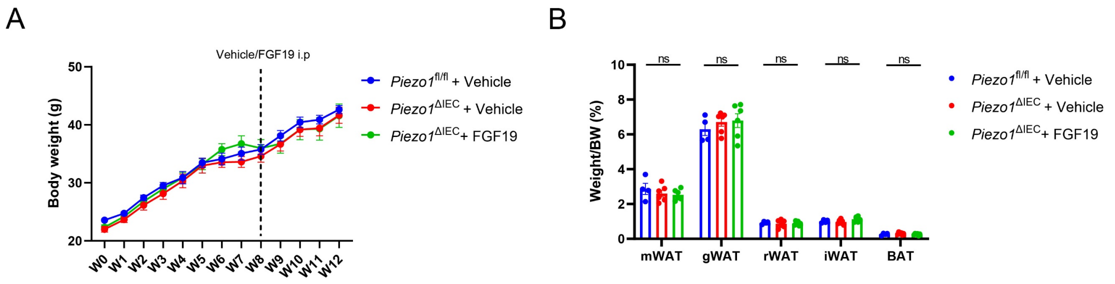

Supplementary Figure 5. Exogenous FGF19 did not alter weight of body or adipose tissues on *Piezo1*$^{\Delta IEC}$ mice

(A) Body weight during 12-week HFD. (B) Relative weight of white adipose tissue (mesentery, gonad, renal and inguen) and brown adipose tissue, of 3 group mice after feeding with HFD for 12 weeks, n=6/group. Data are presented as mean ± sem. *P < 0.05, **P < 0.01, ***P < 0.001. ns, not significant.

Supplementary Material 2

1. Targeted metabolomics of bile acids (BAs)

(1) Chemicals and Reagents

All BAs standards were purchased from Steraloids (Newport, RI, USA) and TRC Chemicals (Toronto, ON, Canada), as well as synthetized by Metabo-Profile (Shanghai, China). The 10 isotope labeled BAs internal standards were purchased from C/D/N Isotops (Quebec, Canada) and Steraloids (Newport, RI, USA).

Analytical pure ammonium acetate was purchased from Sigma Aldrich (St. Louis, MO, USA). Chromatographic pure methanol, isopropanol, acetonitrile, glacial acetic acid, and formic acid were purchased from Thermo Fisher Scientific (FairLawn, NJ, USA). Ultra-pure water is produced by the Mill-Q ultra-pure water system (Millipore, Billerica, MA) equipped with LC-MS Pak filter membranes.

(2) Sample Preparation

10mg cecal contents, pre cooled grinding beads and 200μl acetonitrile/methanol (v/v=8:2) mixed solvent containing 10μl internal standards were added into a centrifuge tube. After homogenization, the tube was centrifuged at 13500 rpm for 20 minutes at 4°C. 10μl supernatant was diluted with 90μl mixed solution composed by acetonitrile/methanol (80/20) and ultrapure water (1:1). After shaking and centrifuging, 5μl solution was injected for analysis.

(3) Instrumentation

Ultra-high performance liquid chromatography tandem mass spectrometry (UPLC-MS/MS, ACQUITY UPLC-Xevo TQ-S, Waters Corp., Milford, MA, USA) was used to quantify BAs concentration in cecal contents. The optimized instrument settings are briefly described below.

**UPLC-MS/MS instrument settings**	
**UPLC**	
Column	ACQUITY UPLC Cortecs C18 1.6 μM VanGuard pre-column (2.1×5 mm)<br>ACQUITY UPLC Cortecs C18 1.6 μM analytical column (2.1 × 100 mm)
Column Temp (°C)	30

Sample Manager Temp (°C)	10
Mobile Phases	A=water with formic acid (pH =3.25); and B=acetonitrile / methanol (80:20)
Gradient Conditions	0-1 min (5% B), 1-3 min (5-30% B), 3-15 min (30-100% B), 15-16 min (100-5%B), 16-17 min (5%B)
Flow Rate (mL/min)	0.40
Injection Vol (μl)	5.0
**Mass Spectrometer**	
Capillary (Kv)	2.0 (ESI-)
Ion Source Temp (°C)	150
Desolvation Temp (°C)	550
Desolvation Gas Flow (L/Hr)	1000

(4) Quality control and analysis of data

Three types of quality control samples including test mixtures, internal standards, and pooled biological samples are routinely used. Raw data was processed by QuanMET software v1.0 (Metabo-Profile, Shanghai, China), which established standard curves, and quantifies each BAs.

2. Measurement of retinol and all-trans retinoic acid

(1) Chemicals and Reagents

Mass spectrometry pure methanol, acetonitrile, formic acid and hexane were purchased from Thermo Fisher Scientific (FairLawn, NJ, USA). Analytical pure ammonium acetate was purchased from Sigma Aldrich (St. Louis, MO, USA). Ultra-pure water is produced by the Mill-Q ultra-pure water system (Millipore, Billerica, MA) equipped with LC-MS Pak filter membranes. Standard mother liquor was diluted with 50% methanol solution to obtain standard samples in different concentration and make the standard curve.

(2) Sample Preparation

15mg ileum and 5μl deionized water were mixed to homogenize. Then 100μl working fluid containing internal standards were added and homogenized for 10 minutes. Sucked 100μl supernatant, transferred to a 2ml glass vial, and then added 10μl hydrochloric acid (4mol/L). After capping, vibrated the vial for 30 seconds, added 800μl hexane, placed on a thermostatic oscillator and extracted at 2000rpm for 15

minutes at room temperature (MSC-100，Allsheng Instruments Co.,Ltd., Hangzhou, China). After centrifuging at 4000g for 20 minutes, 690μl supernatant was transferred to 2ml vial and air-dried. After dissolved by 50μl acetonitrile, the sample was be ready for test and analysis.

(3) Instrumentation

Ultra-high performance liquid chromatography tandem mass spectrometry (ACQUITY-I Xevo TQ-S, Waters Corp., Milford, MA, USA) was used to quantify retinol and all-trans retinoic acid concentration in ileum.

**UPLC-MS/MS instrument settings**	
**UPLC**	
Column	Atlantis Premier BEH C18 AX 1.7 μM analytical column (2.1 × 100 mm)
Column Temp (°C)	40
Sample Manager Temp (°C)	10
Mobile Phases	A=0.1% formic acid; and B=0.1% formic acid methanol
Gradient Conditions	0-2min (0-80% B), 2-3min (80% B), 3-4min (80-90%B), 4-5.5 min (90% B), 5.5-7 min (90-100% B), 7-9.5min (100%B), 9.5-11min (100-0%B),11-12min (0%B)
Flow Rate (mL/min)	0.3
Injection Vol (μl)	10
**Mass Spectrometer**	
Capillary (Kv)	3.0 (ESI+)
Ion Source Temp (°C)	150
Desolvation Temp (°C)	500
Desolvation Gas Flow (L/Hr)	1000

(4) Quality control and analysis of data

The samples were thawed slowly on the ice bath, and the reagent used for extraction was pre-frozen and stored in the refrigerator at -20°C. Whole sample preparation process was completed as soon as possible. There were reagent blank samples and mixed quality control samples before and after the sample analysis of each batch. Raw data was processed by MassLynx software (v4.1, Waters, Milford, MA,USA) to quantifies retinol and all-trans retinoic acid.

3. Real-time quantitative polymerase chain reaction (RT-qPCR)

	**Forward (5'-3' sequence)**	**Reverse (5'-3' sequence)**
**Mouse**		
Gapdh	AGGTCGGTGTGAACGGATTTG	TGTAGACCATGTAGTTGAGGTCA
Piezo1	AGGTGGTCAGTGTTGATGCC	ATTCCGCCCTCATCAAGTGG
Srebp1c	GATGTGCGAACTGGACACAG	CATAGGGGGCGTCAAACAG
Acly	ACCCTTTCACTGGGGATCACA	GACAGGGATCAGGATTTCCTTG
Acc1	CAGTAACCTGGTGAAGCTGGA	GCCAGACATGCTGGATCTCAT
Acc2	CCTTTGGCAACAAGCAAGGTA	AGTCGTACACATAGGTGGTCC
Fasn	AAGCGGTCTGGAAAGCTGAA	AGGCTGGGTTGATACCTCCA
Scd1	TTCTTGCGATACACTCTGGTGC	CGGGATTGAATGTTCTTGTCGT
Elovl6	GAAAAGCAGTTCAACGAGAACG	AGATGCCGACCACCAAAGATA
Dgat1	TCCGTCCAGGGTGGTAGTG	TGAACAAAGAATCTTGCAGACGA
Dgat2	GCGCTACTTCCGAGACTACTT	GGGCCTTATGCCAGGAAACT
Hmgcr	AGCTTGCCCGAATTGTATGTG	TCTGTTGTGAACCATGTGACTTC
Cidea	TGACATTCATGGGATTGCAGAC	GGCCAGTTGTGATGACTAAGAC
Cd36	CCAAATGAAGATGAGCATAGGACAT	GTTGACCTGCAGTCGTTTTGC
Fabp1	ATGAACTTCTCCGGCAAGTACC	CTGACACCCCCTTGATGTCC
Fabp2	GTGGAAAGTAGACCGGAACGA	CCATCCTGTGTGATTGTCAGTT
Fatp2	GATGCCGTGTCCGTCTTTTAC	GACTTCAGACCTCCACGACTC
Il-1β	GAAATGCCACCTTTTGACAGTG	TGGATGCTCTCATCAGGACAG
Il-6	TAGTCCTTCCTACCCCAATTTCC	TTGGTCCTTAGCCACTCCTTC
Tnfα	CCCTCACACTCAGATCATCTTCT	GCTACGACGTGGGCTACAG
Ccl2	CAGCAGGTGTCCCAAAGAAG	TTCCGATCCAGGTTTTTAAT
Ccl3	TGTACCATGACACTCTGCAAC	CAACGATGAATTGGCGTGGAA
F4/80	AATCGCTGCTGGTTGAATACAG	CCAGGCAAGGAGGACAGAGTT
Npc1l1	TGTCCCCGCCTATACAATGG	CCTTGGTGATAGACAGGCTACTG
Mogat2	TGGGAGCGCAGGTTACAGA	CAGGTGGCATACAGGACAGA
ApoA1	AGCAAGATGAACCCCAGTCC	CACATAGTCTCTGCCGCTGT
ApoB	TTGGCAAACTGCATAGCATCC	TCAAATTGGGACTCTCCTTTAGC
Mttp	AGCCAGTGGGCATAGAAAATC	GGTCACTTTACAATCCCCAGAG
Abcg5	AGGGCCTCACATCAACAGAG	GCTGACGCTGTAGGACACAT
Abcg8	CTGTGGAATGGGACTGTACTTC	GTTGGACTGACCACTGTAGGT
Zo-1	GGGAAAACCCGAAACTGATG	GCTGTACTGTGAGGGCAACG
Occludin	CCCAGGCTTCTGGATCTATGT	TCCATCTTTCTTCGGGTTTTCA
Fxr	TCCACAACCAAGTTTTGCAG	TCTCTGTTTGTTGTACGAATCCA
Shp	TCTGCAGGTCGTCCGACTATTC	AGGCAGTGGCTGTGAGATGC
Fgf15	GAGGACCAAAACGAACGAAATT	ACGTCCTTGATGGCAATCG

Cyp7a1	AACAACCTGCCAGTACTAGATAGC	GTGTAGAGTGAAGTCCTCCTTAGC
Cyp8b1	ACAGCGTGATGGAGGAGAGT	AGGGGAAGAGAGCCACCTTA
Cyp7b1	TGAGGTTCTGAGGCTGTGC	TGGAGGAAAGAGGGCTACAA
Cyp27a1	CCAGGCACAGGAGAGTACG	GGGCAAGTGCAGCACATAG
Lrat	GGAACAACTGCGAACACTTTG	CCAGACATCATCCACAAGCA
Adh1	AAGACTACAGCAAACCCATCC	GACGACGCTTACACCACAT
Adh6a	GTGCAAGTTGAGCCACCGA	CACCACGAGTTCTCCTTTCAG
Rdh1	GTCATGGGCCGAATGTCTTTC	CACAAGTCTTGAAGCCTCCAG
Rdh7	TGGGTCGAGTGTCTTTGTGTG	AACCCGCCAGGCTCTATGATA
Rdh10	GAACATCGTAGTGGAGTTCTTCG	CGGTCTCCTCATTGCTCTGC
Rdh13	ATACCTGGGAAGACTGTTATCGT	TGACCAGAATGTCTACTCGCT
Aldh1a1	CTCCTGGCGTGGTAAACATT	CCATGGTGTGCAAACTCAAC
Aldh1a2	TGGGTGAGTTTGGCTTACGG	AGAAACGTGGCAGTCTTGGC
Aldh1a3	ATCAAACCCACGGTCTTCTC	TTTGTCCAGGTTTTTGGTGA
Human		
GAPDH	GTCTCCTCTGACTTCAACAGCG	ACCACCCTGTTGCTGTAGCCAA
PIEZO1	GGACTCTCGCTGGTCTACCT	GGGCACAATATGCAGGCAGA
FXR	GACTTTGGACCATGAAGACCAG	GCCCAGACGGAAGTTTCTTATT
SHP	TTCAACCCCGATGTGCCAG	AAGAAGGCCAGCGATGTCAA
FGF19	ACATGCCTCCCCATGGATTG	AAGTGCTGGGGAAAGGGTTC
